# Tolerability of IM penicillin G benzathine diluted or not with local anesthetics, or different gauge needles for syphilis treatment: a randomized clinical trial

**DOI:** 10.1186/s12879-019-4490-5

**Published:** 2019-10-23

**Authors:** Vicente Estrada, Eva Santiago, Inmaculada Cabezas, Juan Luis Cotano, Juan Carlos Carrió, Manuel Fuentes-Ferrer, Mar Vera, Oskar Ayerdi, Carmen Rodríguez, Laura López, Noemí Cabello, María José Núñez, Teresa Puerta, Iñigo Sagastagoitia, Jorge Del Romero

**Affiliations:** 1 Medicina Interna/enfermedades infecciosas, Hospital Clínico San Carlos, IdiSSC, Universidad Complutense, c/Martin Lagos SN, 28040 Madrid, Spain; 2 Centro Sanitario Sandoval, IdiSSC, C/Sandoval, 7, 28010 Madrid, Spain

**Keywords:** Syphillis, Penicillin G Benzathine, Intramuscular injections

## Abstract

**Background:**

Penicillin G Benzathine (PGB) is the cornerstone of syphilis treatment. However, its intramuscular (IM) administration is associated with pain at the site of injection. The dilution of PGB with local anesthetics is recommended in some guidelines, but the evidence that supports it, particularly in adults and in HIV infection, is scarce. Preliminary clinical experience also suggests that the IM administration of PGB through increased needle gauges might improve its tolerability. The aim of the study to identify less painful ways of administering IM PGB in the treatment of syphilis in adults.

**Methods:**

Multicenter, randomized, double-blinded clinical trial in patients diagnosed with primary syphilis that required a single IM injection of PGB 2400,00 IU. Patients were randomized to receive PGB diluted with 0.5 mL mepivacaine 1% (MV) or PGB alone, and both groups either with a long 19G or short 21G IM needle. The primary objective was the effect on local pain immediately after the administration through a visual scale questionnaire on pain (0 to 10).

**Results:**

One hundred eight patients were included, 27 in each group. Ninety-four (94.4%) were male, and 41.7% were also HIV-infected. Mean age 36.6 years (SD 11). Significant differences in immediate pain intensity were observed when comparing the long 19G group with anesthesia (mean pain intensity, [MPI] 2.92 [CI 95% 1.08-4.07]) vs long 19G without anesthesia (MPI 5.56 [CI 95% 4.39-6.73), *p* < 0.001; and also between short 21G group with anesthesia (MPI 3.36 [CI 95% 2.22-4.50]) vs short 21G without anesthesia (MPI 5.06 [CI 95% 3.93-6.19]), *p* = 0.015). No significant differences in immediate pain were observed between 19G and 21G in the presence or absence of anesthesia (*p* = 1.0 in both cases). No differences were found between study arms after 6 and 24 h.

**Conclusions:**

The IM administration of 1% mepivacaine-diluted PGB induces significantly less immediate local pain as compared to PGB alone. The needle gauge did not have any effect on the pain. Based on these results, we suggest anesthetic-diluted IM PGB as the standard treatment for primary syphilis.

**Trial registration:**

EudraCT 2014-003969-24 (Date of registration 18/09/2014).

## Background

Intramuscular (IM) Penicillin G Benzathine (PGB) is the cornerstone of the treatment of primary syphilis [1], but its administration is associated with sharp pain at the site of the injection that sometimes may be of severe intensity. Some treatment guidelines recommend the administration of PGB diluted with local anesthetics [2], but the evidence behind that, especially in adults and in HIV infection, is scarce. Amir et al. [3] compared in a randomized, double-blind, crossover trial in 18 children the administration of PGB with two diluents, lidocaine hydrochloride 1% versus sterile water. Use of lidocaine hydrochloride as a diluent for PGB did not change the penicillin concentration and significantly reduced the pain of injection. There are no studies on the efficacy and safety of this practice in adults and when PGB is used in the treatment of syphilis. Because of the hypothetical possibility that the crystallization of the drug during its administration had some relation with the intensity of the pain, we hypothesized that the administration of PGB through a wider and longer needle could induce less pain. Our study aims to identify less painful ways of administering PGB in the treatment of syphilis in adults, in particular, diluted together with local anesthetic and with needles of a different gauge.

## Methods

### Study design

**Fig. 1:**
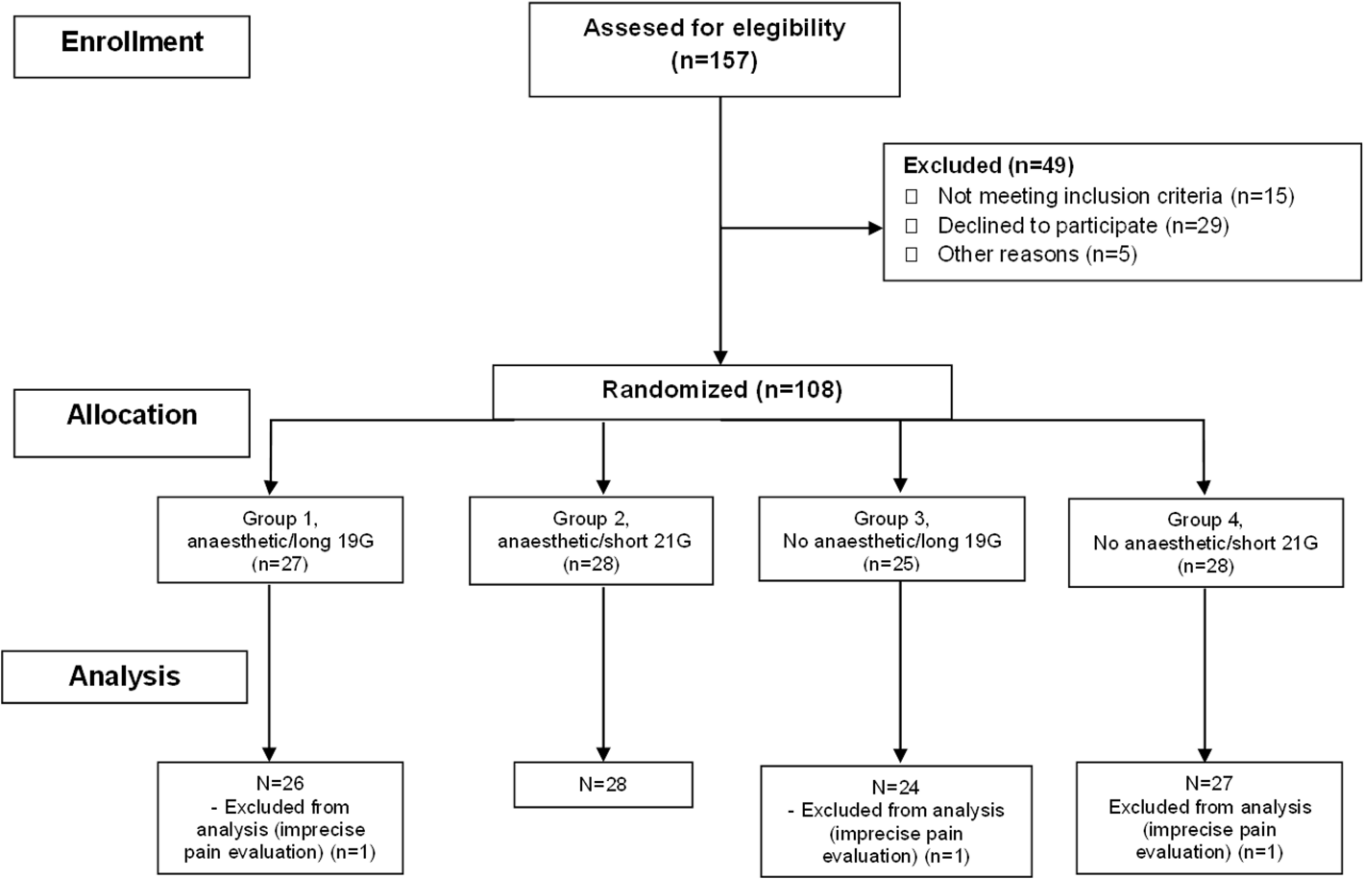
Consort flow diagram of the study

We conducted a randomized, double-blind clinical trial at two sites in Madrid (Centro Sanitario Sandoval and Hospital Clinico San Carlos, to compare the effect on immediate pain of PGB diluted with a local anaesthetic (mepivacaine hydrochloride 1%, MV) or PGB alone, and both groups either with a long 19G (long) or short 21G (short) IM needle. The study was performed during the first half of 2015. All patients signed informed consent. The study was approved by the local institutional review board (Ethical Committee of Clinical Research, Health Research Institute of the Hospital Clínico San Carlos) with approval number CEIC#14/376, and registered at the EU Clinical Trials Register with EudraCT Number: 2014-003969-24. The Consort flow diagram of the study is presented in Fig. 1.

### Participants

Eligible subjects were 18 years of age or older diagnosed with primary syphilis; this diagnosis was based on both clinical and positive serological test results (non-treponemal and treponemal tests). Syphilis treatment required one IM dose of PGB 2400,000 million units (PGB 2,4 MU). We excluded patients with any sensory perception diagnosis or cognitive impairment unable to understand or evaluate the visual analog score on pain. Patients with previous penicillin allergy were also excluded.

### Intervention

PGB was diluted with 6 mL of sterile water as indicated in information pamphlet (Benzetacil 2.400.00 U/6 mL, Reig Gofre, Barcelona, Spain); when MV was added to PGB, it was diluted with 5.5 mL of sterile water. A 0.5 mL dose of 1% MV was used to dilute with PGB; this corresponds to a total dose of 5 mg of MV. The study nursing team was trained to administer IM in the upper-outer quadrant of the buttock in the same way. Patients were randomly assigned to one of the four following groups: group 1, anesthetic/long 19G needle; group 2, anesthetic/short 21G needle; group 3, no anesthetic/long 19G needle; group 4, no anesthetic/short 21G needle.

### Randomization and masking

The study participants were randomly assigned to study groups by a computer-generated randomized number system in blocks. The randomization was done 1:1:1:1 in blocks of four. The randomization sequence was hidden for the investigators who made the selection of patients. The design of the study was double-blind: neither the patient nor the investigator who analyzed the pain results knew the assigned group.

### Outcomes

The primary objective of the study was the effect on local pain immediately after the injection; patients indicated the intensity of pain on a visual scale enumerated from 0 (no pain) to 10 (maximum pain) just after IM injection. Secondary objectives were pain intensity 6 and 24 h after the IM injection; this information was obtained by a phone call. Information was also collected on HIV status, age, sex, race, and body mass index (BMI).

### Statistical analysis

#### Sample size calculation

In the absence of any published study regarding the effect of needle gauge on pain, we estimated the sample size with data from a convenience sample of consecutive patients with syphilis treated with one dose of IM PGB 2.4 MU. We studied immediate pain after the injection, in 22 patients distributed in the same four groups; we assessed needle gauge and the effect of anesthetic with a visual pain score (0-10). We compared 19G with anesthesia vs. 21G with anesthesia; 19G without anesthesia vs. 21G without anesthesia; and 19G with anesthesia vs. 19G without anesthesia. The estimation of the global sample size was based on the larger sample required for comparisons. Based on expected mean pain intensity of 7.6 in the 19G group without anesthesia and 8.6 the 21G group without anesthesia, with a significance level of 1.7% (Bonferroni adjustment 0.05/3) and statistical power of 80%, we considered that 104 patients (26 in each group) were needed.

Qualitative variables were summarized by their frequency distribution and quantitative variables by their mean and standard deviation (SD). Baseline participant characteristics were reported according to the randomly assigned intervention group with the use of descriptive statistics. To evaluate the differences in the intensity of the pain (immediate, 6 and 24 h after the injection) between the study groups, a multiple linear mixed model was fitted for controlling the hospital-cluster effect with adjustment for BMI. All analyses were based on intention to treat randomization status, and the statistical significance was assessed at the Bonferroni-corrected for multiple testing (α/3 = 0.017). All analyses were performed using STATA 12.0 software.

## Results

**Fig. 2:**
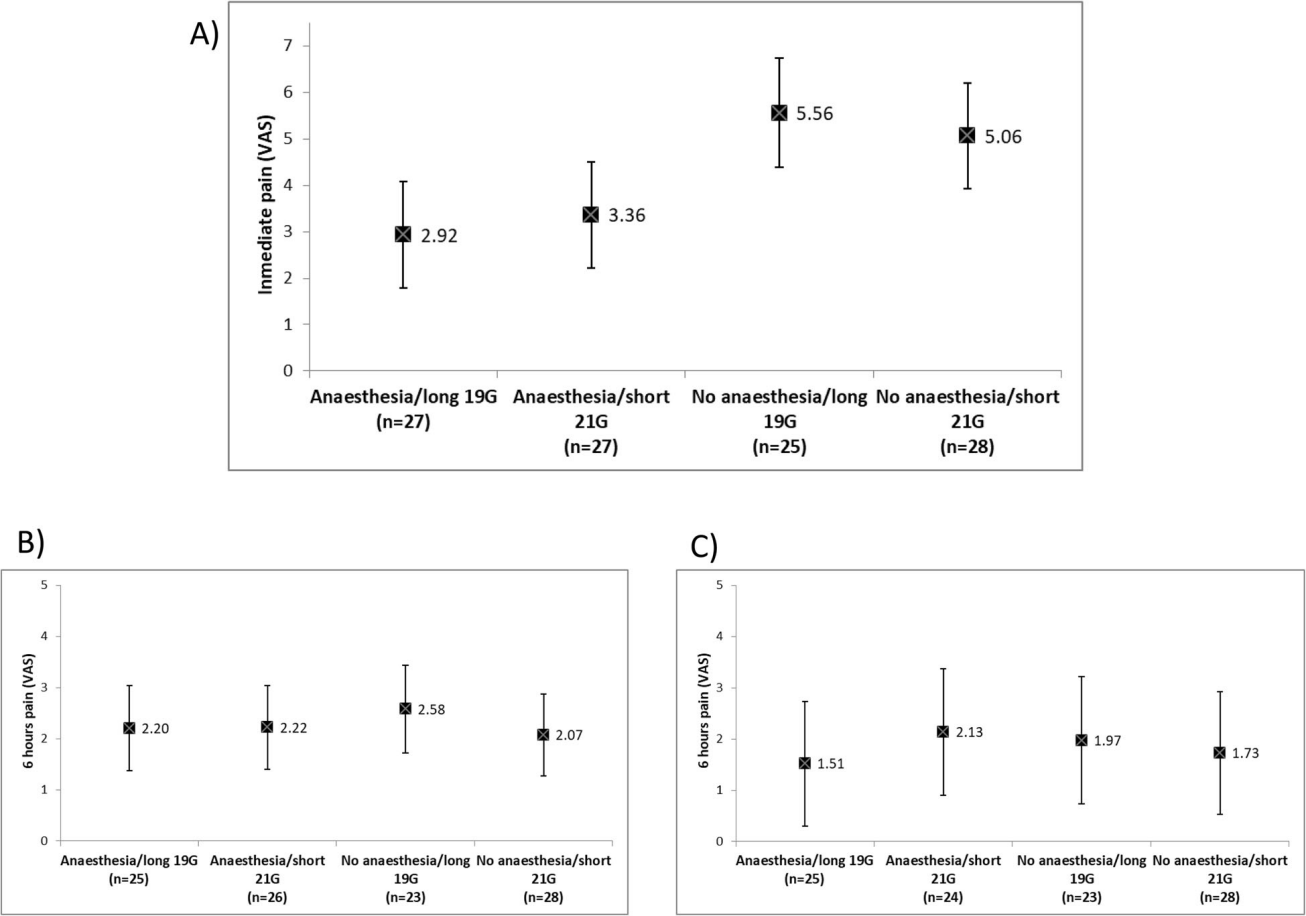
Differences in pain intensity between groups; **a** immediate pain; **b** 6 h after the IM injection; and **c** 24 h after the IM injection. Statistically significant differences on pain intensity were found between anesthesia and no-anesthesia groups regardless of the needle size (long 19G with anesthesia vs. long 19G without anesthesia, *p* < 0.001; and short 21G with anesthesia vs. short 21G without anesthesia, *p* = 0.015)

Patient characteristics are shown in Table 1. In summary, study participants were mostly Caucasian men with normal BMI, and 41% also with HIV-infection. Significant differences in immediate pain intensity were observed when comparing the long 19G group with anesthesia (mean pain intensity, [MPI] 2.92 [CI 95% 1.08-4.07]) vs long 19G without anesthesia (MPI 5.56 [CI 95% 4.39-6.73), *p* < 0.001; and also between short 21G group with anesthesia (MPI 3.36 [CI 95% 2.22-4.50]) vs short 21G without anesthesia (MPI 5.06 [CI 95% 3.93-6.19]), *p* = 0.015). No significant differences in immediate pain were observed between 19G and 21G in the presence or absence of anesthesia (*p* = 1.0 in both cases). There were no significant differences in pain intensity between groups at 6 and 24 h. These results are shown in Fig. 2.

**Table 1:** Clinical characteristics of the study population

	Group 1, anaesthesia /long 19G needle *n* = 27	Group 2, anaesthesia /short 21G needle *n* = 28	Group 3, no anaesthesia/long 19G needle *n* = 25	Group 4, no anaesthesia/short 21G needle *n* = 28	Total *n* = 108
Gender male (%)	27 (100)	26 (92.9)	23 (92)	26 (92.9)	102 (94.5)
Age (years)	34.2 (10.5)	36.1 (8.7)	39.0 (10.6)	37.5 (14.1)	
BMI (kg/m^2^), SD	23.8 (3.8)	24.9 (4.9)	25.6 (3.9)	23.9 (2.8)	
Ethnicity (%)					
Caucasic	17 (63)	22 (78.6)	18 (72)	22 (78.6)	79 (73.1)
Latin	7 (25.9)	6 (21.4)	5 (20)	5 (17.9)	23 (21.3)
Black	1 (3.7)	0 (0.0)	2 (8.0)	1 (3.6)	4 (3.7)
Other	2 (7.4)	0 (0.0)	0 (0.0)	0 (0.0)	2 (1.8)
HIV infection	12 (44.4)	12 (42.9)	12 (48)	9 (32.1)	45 (41.7)
Center					
Sandoval	19 (70.4)	20 (71.4)	18 (72)	19 (67.9)	76 (70.4)
HCSC	8 (29.6)	8 (28.6)	7 (28)	9 (32.1)	32 (29.6)

## Discussion

The severity of pain after the IM administration of PGB may reduce the adherence to treatment and thus, improvements in tolerability might improve treatment compliance [4]. Other studies have shown that the use of local anesthetics together with other drugs administered IM for other indications, for example, lidocaine together with kanamycin [5] or amikacin [6], significantly reduces pain immediately after injection and does not reduce the effectiveness of the treatment. In another study, the administration of two IM doses of 1.2 MU together with 0.5 mL lidocaine did not show differences in pain or patient preference, compared to the standard dosing of single 2.4 MU without anesthesia, in patients with syphilis treated with three doses of PGB [7].

Our study shows that the administration of PGB diluted with a local anesthetic significantly reduces the intensity of pain immediately after intramuscular injection. These results are similar to those observed in a pediatric population treated with PGB for a different indication of syphilis [3], support the recommendations of international treatment guidelines [2], and confirm the daily clinical experience. However, the hypothesis that the administration with a larger and longer gauge could further reduce pain is not confirmed by our results. We based this hypothesis on the fact that PGB can crystallize in the same syringe, and that it could be in part responsible of the pain due to muscle damage; also, it was based in a small exploratory study sample of patients in which the size of the needle was associated to a reduced pain intensity. In our study, we did not find that ethnicity, HIV status or BMI influences the response to pain after the IM administration of PGB with local anesthetics or different gauge needles. The effect of anesthesia is only immediate since no differences between groups were found in the results in pain intensity beyond that time point.

The main strength of our study is the confirmation with a double-blind clinical trial, with adequate statistical power, of the efficacy of the PGB administered together with anesthetic in the pain control. We can, therefore, confirm that PB diluted with anesthetic significantly reduces the pain of IM administration of PGB. A limitation of our study is that most of our patients were young males (94%), and these results may not be similar in women or patients of different ages.

## Conclusions

The administration of PGB diluted with a local anesthetic significantly reduces the intensity of pain immediately after intramuscular injection; we did not find that the use of larger or longer needles exerts any influence on immediate pain. We suggest administering local anesthetic-diluted PGB as primary syphilis therapy.

## Data Availability

The datasets used and analysed during the current study are available from the corresponding author on reasonable request.

## Abbreviations

IM: Intramuscular
MPI: Mean pain intensity
MV: Mepivacaine hydrochloride
PGB: Penicillin G Benzathine
RN: Registered Nurse
SD: Standard deviation
